# Highly Stretchable, Low Hysteresis, and Transparent Ionogels as Conductors for Dielectric Elastomer Actuators

**DOI:** 10.3390/gels11050369

**Published:** 2025-05-17

**Authors:** Limei Zhang, Hong Li, Zhiquan Li, Weimin Pan, Yi Men, Niankun Zhang, Jing Xu, Xuewei Liu

**Affiliations:** 1 School of Exercise and Health Sciences, Xi’an Physical Education University, Xi’an 710068, China; 2 Engineering Research Center of Innovative Technology of Intelligent Sports Equipment, Universities of Shaanxi Province, Xi’an 710068, China; 3 School of Mechanical Engineering, Xi’an Aeronaut University, Xi’an 710077, China; 4 School of Mechanical and Electrical Engineering, Xi’an University of Architecture and Technology, Xi’an 710055, China

**Keywords:** ionogel, stretchability, transparency, low hysteresis, actuator, soft electronics

## Abstract

As conductive materials, ionogels have attracted significant attention for their potential applications in flexible wearable electronics. However, preparing an ionogel with mechanical properties akin to human skin while also achieving transparency, adhesion, and low hysteresis through simple processes remains challenging. Here, we introduce a multifunctional ionogel synthesized via a one-step photopolymerization method. By leveraging the good compatibility between the ionic liquid and the polymer network, as well as the hydrogen bonding and chemical crosslinking within the gel network, we achieved an ionogel with high transparency (>98%), stretchability (fracture strain of 19), low hysteresis (<5.83%), strong adhesion, robust mechanical stability, excellent electrical properties, a wide operating temperature range, and a tunable modulus (1–103 kPa) that matches human skin. When used as a conductor in soft actuators, the ionogel enabled a large area strain of 36% and a fast electromechanical conversion time of less than 1 s. The actuator demonstrated good actuation performance with voltage and frequency dependence, electrochemical stability, and outstanding durability over millions of cycles. This study provides a simple and effective method to produce multifunctional ionogels with tailored mechanical properties that match those of human skin, paving the way for their application in flexible wearable electronics.

## 1. Introduction

Stretchable conductive materials are essential for developing intelligent flexible wearable electronics, with broad applications in electronic skin, human motion detection, human-machine interfaces, and flexible energy storage devices [1,2,3,4]. Over the past few decades, significant progress has been made in the development of both electronic and ionic conductive materials. Electronic conductors transport electric currents through the movement of electrons. Typically, stretchable electronic conductors are fabricated by integrating electron-conducting fillers, such as metals, carbon-based materials, or conductive polymers, into a stretchable elastic matrix via molding, attaching, or encapsulation [5,6,7]. However, these materials often face several limitations, including high production costs, complex manufacturing processes, inherent opacity due to the filler materials, and most critically, the vulnerability of conductive pathways to damage under mechanical strain or cyclic loading [8]. In contrast, ionic conductors transport electric currents via mobile ions, mimicking the mechanisms found in living organisms. Various stretchable ionic conductors have been developed based on polymer networks that incorporate mobile ions and ensure that ion transportation pathways remain intact even when stretched [9]. These stretchable ionic conductors exhibit superior deformation tolerance and are thus more suitable for soft electronics applications.

Hydrogels, a class of ionic conductors [10], possess stretchability, ionic conductivity, and optical transparency, making them promising for applications in sensors [11], artificial muscles [12], display screens [13], and beyond. However, a critical limitation of hydrogel-based conductors is their susceptibility to water evaporation or freezing due to the inherent presence of liquid water, which significantly hinders their practical utility. While incorporating salts into hydrogels can improve water retention, this approach fails to fully prevent dehydration, especially under elevated temperatures [14,15,16]. Since the liquid water content directly governs the electrical and mechanical properties of hydrogels [17], its evaporation or freezing can severely compromise their stability and long-term performance in real-world applications.

Ionogels, another type of ionic conductive material, are swollen with ionic liquids instead of water [18,19,20]. By combining the characteristics of ionic liquids and polymer networks, ionogels exhibit negligible volatility, high ionic conductivity, chemical inertness, thermal stability, and stretchability [21]. The mechanical and electronic properties of ionogels can be tailored by adjusting the ratio of polymer monomer to ionic liquid, enabling them to meet diverse application requirements. Therefore, ionogels have emerged as ideal conductive materials for flexible electronics [22]. To extend their service life, most researchers have focused on enhancing the mechanical properties of ionogels. Various synthetic strategies have been explored, including topological engineering, non-covalent crosslinking, phase-separated structures, and nanocomposite hybridization [23,24,25,26]. These approaches have significantly increased the Young’s modulus of ionogels to over 100 kPa, far exceeding that of human skin (5–100 kPa) [27,28,29,30]. However, this stiffness mismatch between ionogel-based devices and biological tissues can compromise skin conformability and user comfort. Recently, Zheng et al. developed an amino-acid-based ionogel by radical polymerization of N-acryloylglycine in 1-ethyl-3-methylimidazolium ethyl sulfate ([C_2_mim][EtSO_4_]) [31]. The resulting material demonstrated a tunable Young’s modulus (60–70 kPa), closely matching that of human skin, along with strong adhesion due to multiple hydrogen bonds. Nevertheless, it exhibits undesirable yellow coloration and significant hysteresis. Achieving a combination of high transparency, low hysteresis, and strong adhesion while ensuring that the mechanical properties match those of human skin in a simple and efficient manner remains a challenge for ionogels.

Herein, we introduce a multifunctional polyacrylic acid (PAA) ionogel synthesized via a facile one-step radical polymerization process. The ionogel was fabricated by polymerizing acrylic acid (AA) in the presence of [C_2_mim][EtSO_4_] ionic liquid. The resulting ionogel exhibits high stretchability (fracture strain of 19) and low hysteresis (<5.83%, with dissipated energy of only 1.58 kJ m^−3^), significantly outperforming previously reported PAA- or [C_2_mim][EtSO_4_]-based ionogels (which show fracture strains of 500–1200% and dissipated energies of 2–7.9 × 10^3^ kJ m^−3^) [31,32,33,34,35,36,37]. Moreover, the Young’s modulus of the PAA ionogel (1–103 kPa) closely matches that of human skin and can be finely tuned by adjusting the content of monomers and crosslinkers. The PAA ionogels also exhibit high transparency (>98%), strong adhesion, good mechanical stability, excellent electrical properties, and a wide operating temperature range. Subsequently, the PAA ionogel was successfully demonstrated as an ionic conductor in dielectric elastomer actuators. The actuators showed good actuation performance, with the area strain being dependent on voltage and frequency, and exhibited excellent durability over millions of repeated excitation cycles. The multifunctional properties of the PAA ionogels offer great advantages for applications in soft robotics, human-machine interfaces, artificial muscle systems, and next-generation wearable technologies.

## 2. Results and Discussion

### 2.1. Preparation of PAA Ionogel

**Figure 1:**
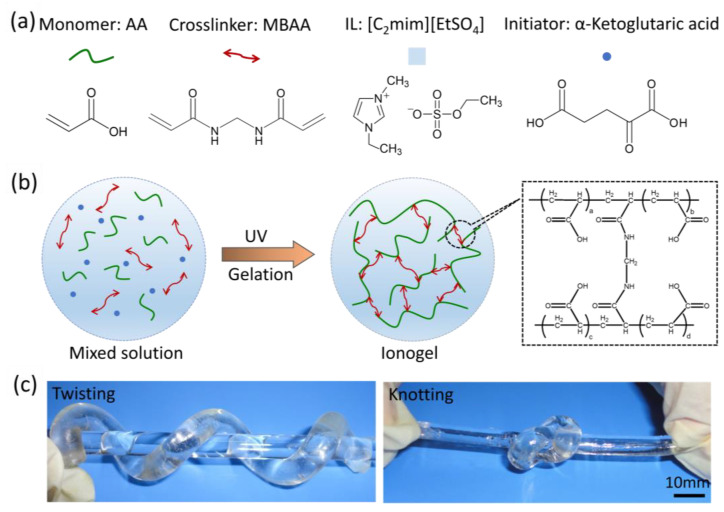
Preparation of the PAA ionogel. (**a**) Chemical structures of the reagents used. (**b**) Schematic illustration of the ionogel preparation process. (**c**) Photographs of the transparent ionogel in twisted and knotted shapes.

The PAA ionogels are fabricated through a one-step radical polymerization method. To begin with, a uniform precursor solution is meticulously prepared, which includes AA as the monomer, N,N′-methylenebisacrylamide (MBAA) as the crosslinker, α-ketoglutaric acid as the initiator, and the ionic liquid [C_2_mim][EtSO_4_] as the solvent (Figure 1a). This well-mixed solution is subsequently transferred into a reaction mold and exposed to UV light at room temperature to initiate the radical polymerization process, ultimately yielding a robust polymer network (Figure 1b). In this way, the PAA ionogels are successfully obtained. One of the key advantages of the PAA ionogels lies in their versatile shaping capability, enabled by the injection molding process. Unlike conventional electronic conductive materials that often struggle with conforming to complex and non-planar surfaces, PAA ionogels can be easily and freely molded into various shapes to match intricate geometries. As illustrated in Figure 1c, the transparent ionogel exhibits remarkable mechanical properties. It can be twisted or knotted without sustaining any damage and has the ability to fully recover its original shape once the applied mechanical stress is removed. These results demonstrate the ionogel’s exceptional elasticity, flexibility, and optical transparency, which arise from synergistic mechanisms: (i) the MBAA crosslinking points serve as stress buffers, enhancing mechanical robustness; (ii) the hydrogen bonds, formed between the carboxyl groups of PAA and the ethyl sulfate moieties of [C_2_mim][EtSO_4_], enable dynamic recombination, facilitating network homogenization; and (iii) the high compatibility between the ionic liquid and the PAA polymer network ensures excellent optical clarity.

### 2.2. Mechanical Properties of PAA Ionogel

**Figure 2:**
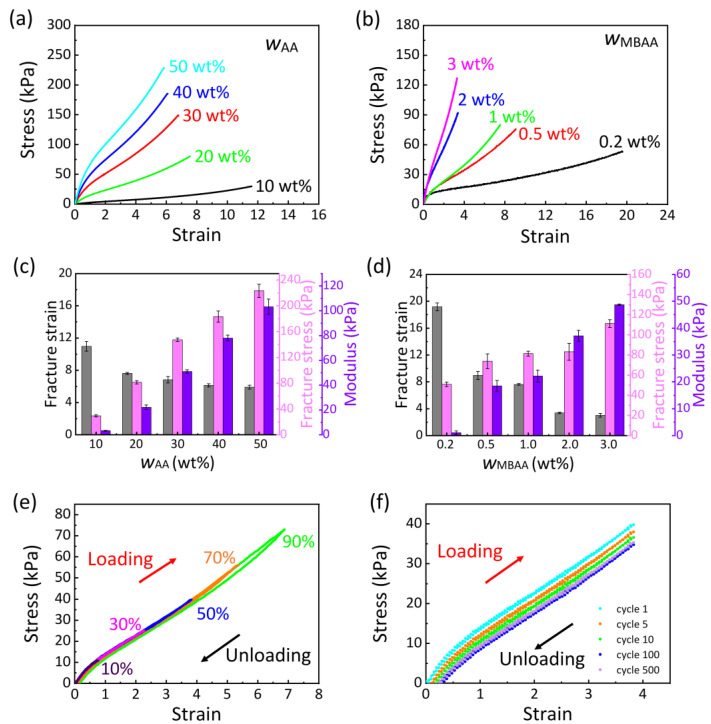
Mechanical properties of PAA ionogel. (**a**) Stress–strain curves of ionogels with various $w_{AA}$ values while $w_{MBAA}$ = 1 wt%. (**b**) Stress–strain curves of ionogels with various $w_{MBAA}$ values while $w_{AA}$ = 20 wt%. Effects of (**c**) $w_{AA}$ and (**d**) $w_{MBAA}$ on fracture strain, fracture stress, and ionogel modulus. (**e**) Cyclic loading–unloading curves of the ionogel at 10%, 30%, 50%, 70%, and 90% of the fracture strain. (**f**) Stress–strain curves of the ionogel over 500 cycles at 50% of the fracture strain. The ionogel with $w_{AA}$ = 20 wt% and $w_{MBAA}$ = 1 wt% is used in (**e**,**f**).

The concentrations of monomer (AA) and crosslinker (MBAA) influence the mechanical properties of PAA ionogels. To systematically investigate these effects, we conducted two distinct series of experiments. In the first series, we varied the weight ratio of AA to ionic liquid ($w_{AA}$) from 10 to 50 wt%, while maintaining the weight ratio of MBAA to AA ($w_{MBAA}$) at a constant 1 wt% (Figure 2a). As $w_{AA}$ increased, the fracture stress and Young’s modulus of the ionogels correspondingly rose, while the fracture strain decreased (Figure 2c). For instance, the ionogel sample with $w_{AA}$ = 10 wt% exhibited the highest fracture strain (10.98 ± 0.6) but the lowest fracture stress (29.27 ± 1.44 kPa) and Young’s modulus (3.26 ± 0.42 kPa). Conversely, the sample with $w_{AA}$ = 50 wt% demonstrated the highest fracture stress (223.26 ± 10.12 kPa) and Young’s modulus (103.3 ± 6.21 kPa), yet the lowest fracture strain (5.91 ± 0.24). At lower $w_{AA}$ values, the ionogels, characterized by their dilute PAA polymers, were highly flexible and could be stretched to large deformations but were unable to withstand large loading forces. In the second series of experiments, we varied the $w_{MBAA}$ from 0.2 to 3 wt%, while keeping $w_{AA}$ constant at 20 wt% (Figure 2b). Similar trends in mechanical properties were observed. As $w_{MBAA}$ increased, the fracture stress and Young’s modulus of the ionogels rose, while the fracture strain decreased (Figure 2d). For example, the sample with $w_{MBAA}$ = 0.2 wt% exhibited the highest fracture strain (19.17 ± 0.57) but the lowest fracture stress (50.83 ± 2.28 kPa) and Young’s modulus (1.1 ± 0.62 kPa). In contrast, the sample with $w_{MBAA}$ = 3 wt% achieved the highest fracture stress (111.27 ± 3.91 kPa) and Young’s modulus (48.71 ± 0.26 kPa), while having the lowest fracture strain (3.01 ± 0.28). At lower $w_{MBAA}$ values, the ionogels with more loosely crosslinked networks and longer PAA chains could be stretched to large deformations but were unable to sustain high loading forces. Overall, all PAA ionogels demonstrated favorable mechanical properties, which can be attributed to the synergistic effects of chemical crosslinking and the hydrogen bonds within the ionogels. Specifically, the resulting ionogels achieved a maximum strain of 19 and a Young’s modulus that could be tuned between 1 kPa and 103 kPa, closely resembling the mechanical properties of human skin. However, samples with low polymer chain density or crosslink density tend to be overly soft and sticky, despite their enhanced stretchability. To balance stretchability and practical operability, we selected the PAA ionogel with a monomer content of $w_{AA}$ = 20 wt% and a crosslinker content of $w_{MBAA}$ = 1 wt% as the subject for subsequent experimental investigations.

The ability of the conductor to undergo elastic deformation is crucial for ensuring precise, reliable, and consistent performance of stretchable devices. To evaluate the elastic deformation capacity of the PAA ionogel, we conducted cyclic loading–unloading tests, gradually increasing the strain (Figure 2e). The PAA ionogels demonstrated very low hysteresis values of 1.15%, 1.37%, 1.89%, 3.43%, and 5.83% at 10%, 30%, 50%, 70%, and 90% of the fracture strain, respectively. These values are significantly lower than those reported for other ionic conductors in the literature [38,39,40,41]. For instance, during the loading–unloading cycle at 50% of the fracture strain (a strain of 3.83), the dissipated energy of the ionogel was only 1.58 kJ m^−3^, which is 1.89% of the total energy (83.5 kJ m^−3^). This minimal energy loss indicates the super-elastic nature of the PAA ionogel. Furthermore, loading–unloading curves were recorded over 500 cycles at 50% of the fracture strain (Figure 2f). In the initial 50 cycles, the maximum stress of each cycle gradually decreased before stabilizing in the subsequent cycles. This initial stress reduction is attributed to structural reconfiguration within the polymer networks, transitioning from a transient to a stable state. This reconfiguration is enabled by the disentanglement of polymer chains. Notably, the hysteresis remained minimal even after thousands of cycles, which can be attributed to the synergistic effects of the covalent crosslinking and hydrogen bonding among PAA, the ionic liquid, and MBAA. These results demonstrate the stable mechanical performance of PAA ionogels and confirm that they are ideal ionic conductors, highly suitable for applications in flexible wearable electronics.

### 2.3. Multifunctional Properties of PAA Ionogel

**Figure 3:**
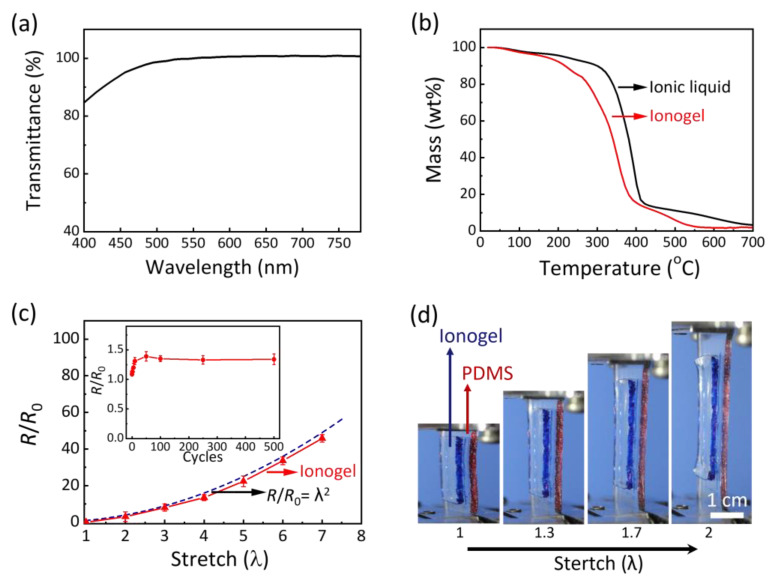
Multifunctional properties of PAA ionogels. (**a**) Transmittance of ionogel in the wavelength range from 400 nm to 780 nm. (**b**) TGA curves of the ionogel and the ionic liquid with a heating rate at 10 °C min^−1^. (**c**) The ratio of the resistance of the deformed ionogel to that of an undeformed sample (*R*/*R*_0_) as a function of the stretch ratio *λ*, where *λ* represents the multiple of the ionogel’s original length. The theoretical predictions, given by the equation *R*/*R*_0_ = *λ*^2^, are in good agreement with the experimental values. The resistance ratio *R*/*R*_0_ is a function of cycle number during cyclic stretching to a strain of 3.83, which corresponds to 50% of the fracture strain (inset). (**d**) An illustration of conformal stretching deformation for the ionogel when integrated with a PDMS sheet.

The ionogel exhibits excellent transparency, characterized by an average transmittance exceeding 98% across the visible spectrum (400 nm to 780 nm, Figure 3a), which demonstrates its potential for applications requiring high light transmission. This exceptional optical performance stems from the good interaction between the PAA polymer network and the [C_2_mim][EtSO_4_] ionic liquid. Such high transparency renders the ionogel an ideal material for use in touchscreens, electronic eyes, and other soft optoelectronic devices where clarity and minimal optical interference are of critical importance. The thermal stability of the ionogel is an important property for its practical applications. Thermogravimetric analysis (TGA) was employed to examine its stability over a wide temperature range. The results indicate that the PAA ionogel can endure temperatures up to 250 °C without significant degradation (Figure 3b). This capability to maintain structural and functional integrity across a broad temperature range enables the ionogel to operate effectively in extreme environmental conditions. The decomposition temperature of the PAA ionogel is lower than that of the pure ionic liquid ([C_2_mim][EtSO_4_]), which decomposes at 330 °C. The earlier mass loss observed in the ionogel is mainly attributed to the breakdown of the PAA polymer network [37]. Moreover, the non-volatile nature of the ionogel ensures its stability and functionality when exposed to open air. This characteristic is particularly important for applications in ambient conditions, where special containment or protective measures are often inconvenient or impractical. The ionogel’s ability to maintain its properties in open-air environments without degradation further enhances its practicality for real-world applications.

The electrical properties of the PAA ionogel were thoroughly investigated. The electrical resistance of the ionogel is closely related to its dimensions. When the ionogel is stretched, its resistance gradually increases due to changes in its geometry. The ratio of the resistance of the stretched ionogel to that of the unstretched sample (*R*/*R*_0_) increases progressively with increasing strain. This observation is in good agreement with the theoretical prediction described by the equation *R*/*R*_0_ = λ^2^ [42] (Figure 3c). Here, *R*_0_ represents the original resistance of the conductor, while *R* denotes the resistance after the conductor is stretched, and *λ* is the ratio of the stretched length to its original length. This finding indicates that the resistivity of the ionogel remains constant during deformation, given that the ionogel is incompressible. To gain a deeper understanding of the electrical behavior of the PAA ionogel under cyclic mechanical stress, we conducted tests to measure the variation of the resistance ratio *R*/*R*_0_ over numerous loading–unloading cycles, with a maximum strain set at 3.83. Initially, during the first 50 cycles, the *R*/*R*_0_ ratio increases before stabilizing in the subsequent cycles (as shown in the inset of Figure 3c). This behavior is consistent with the variations in the stress–strain curve shown in Figure 2f. The initial increase in *R*/*R*_0_ can be attributed to residual strain, which is displayed in Figure 2f. The PAA ionogel exhibits outstanding electrical stability even when subjected to large deformations and repeated stretching cycles, significantly outperforming traditional electronic conductors. For example, carbon nanotube thin films usually experience a dramatic increase in electrical resistance, ranging from 100 to 1000 times, after undergoing hundreds of stretching cycles within a strain range of 0 to 1 [43]. In contrast, our ionogel consistently maintains its electrical properties throughout these tests. The outstanding electrical stability of the PAA ionogel under mechanical deformation is a key factor for its potential practical applications. In flexible and stretchable electronics, components are often subjected to repeated mechanical deformations, which may lead to changes in their electrical properties. The ionogel’s ability to maintain a stable resistance under such conditions suggests that it could be used in flexible wearable electronics, where reliability is of great importance, as well as in those devices that demand high performance under dynamic mechanical conditions. Moreover, the ionogel’s durability implies its potential for long-term use in applications that involve repeated stretching and relaxing. This is especially significant for devices that need to maintain their functionality over extended periods, such as health-monitoring wearables or flexible sensors. The PAA ionogel’s ability to maintain stable electrical properties under mechanical stress, combined with its excellent transparency and thermal stability, makes it a highly promising material for next-generation flexible and stretchable electronics.

In addition, the adhesive characteristics of ionogels also play an important role in the fabrication of the devices. To assess the potential of the PAA ionogel as both an adhesive and a compliant conductor, we examined its bonding capabilities with commonly used elastomer polydimethylsiloxane (PDMS) materials. Our findings revealed that the as-prepared PAA ionogel has strong adhesion and readily forms a strong bond with PDMS surfaces. This robust adhesion is evident when the ionogel is integrated with a PDMS sheet, as it can undergo conformal stretching deformation without delamination (Figure 3d). Even under applied stress, the ionogel and PDMS remain firmly attached, indicating the robustness of their bond. The strong adhesion between the PAA ionogel and PDMS is primarily due to hydrogen bonding between the carboxyl groups of PAA and the PDMS substrate. Although the ionic liquid may also interact with the PDMS substrate, its contribution to the overall adhesive strength is relatively minor. This is because the ionic liquid’s interactions are insufficient to effectively transfer loading to the polymer network, as indicated by the minimal hysteresis loop observed in Figure 2e. Unlike other ionic conductors, the PAA ionogel possesses an inherent stickiness, which significantly contributes to the seamless integration of devices. This intrinsic adhesion ensures that the ionogel can adhere effectively to various substrates, facilitating device assembly and enhancing performance.

### 2.4. PAA Ionogel as Conductors for Dielectric Elastomer Actuators

**Figure 4:**
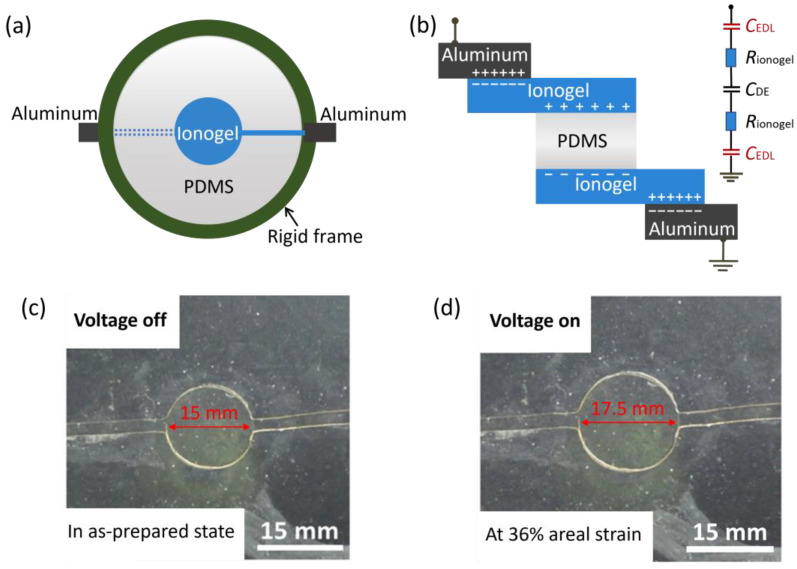
An actuator fabricated by integrating the PAA ionogel with a PDMS dielectric elastomer. Schematic diagrams of the actuator are shown in (**a**) top-down view and (**b**) side view, along with the equivalent circuit of the device. The membrane of the dielectric elastomer is stretched and fixed between two rigid plastic frames. Each face of the dielectric membrane is attached with a layer of the ionogel. Thin lines of the ionogel extend to the rigid frame and meet aluminum wires, which are connected to the power source. (**c**) When the voltage is off, the actuator is in the as-prepared state. (**d**) When a voltage of 20 kV is applied, the actuator expands in area.

We have designed and fabricated a transparent, high-speed, and long-life actuator using PAA ionogel as the electrode and PDMS as the dielectric elastomer. The PDMS film was pre-stretched and secured between two circular rigid plastic frames. Each side of the dielectric membrane was attached with a layer of ionogel, forming a sandwich structure. The ionogel extends to the rigid frame and connects to aluminum tapes, which are linked to the power source. The top-down and side views of this design are shown in Figure 4a,b. The device features a series connection of the power source, aluminum tapes (electronic conductor), ionogel electrode (ionic conductor), and PDMS dielectric elastomer. The aluminum tapes are positioned outside the active region of the device, ensuring both stretchability and transparency. When a voltage was applied, opposite charges accumulate on the two faces of the PDMS dielectric elastomer, forming a capacitance and generating a coulombic force. This force compresses the PDMS dielectric elastomer in the thickness direction while causing it to expand in area (Figure 4c,d). The interface between the ionic electrode and the dielectric elastomer forms an electrical double layer (EDL), which enables effective capacitive coupling into the active layer [44]. The equivalent circuit is illustrated in Figure 4b.

**Figure 5:**
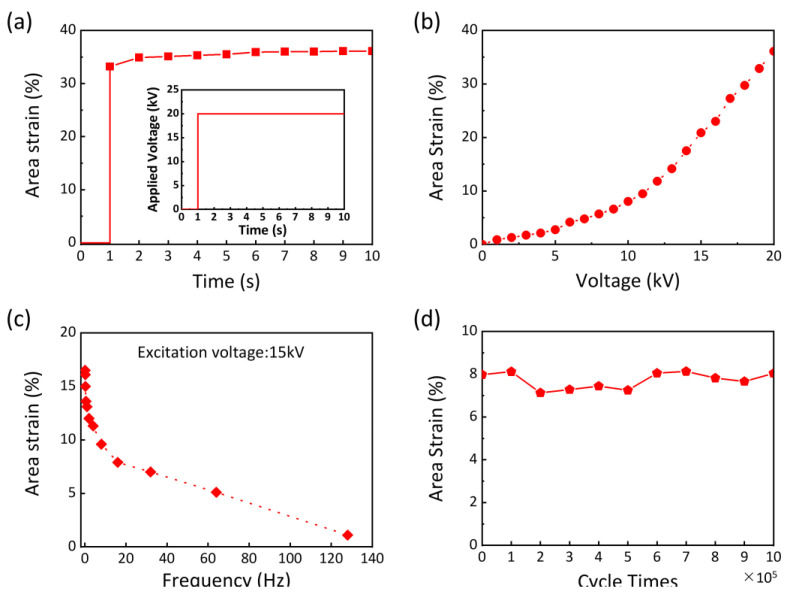
Electromechanical performance of the dielectric elastomer actuator. (**a**) Area strain response to a step voltage input (20 kV applied at 1 s, inset: voltage–time curve). (**b**) Voltage-dependent area strain. (**c**) Frequency-dependent area strain. (**d**) Cyclic reliability test of the actuator over 10^6^ cycles under continuous sinusoidal excitation (15 kV amplitude, 16 Hz frequency).

The modulus of the PAA ionogel is in the order of ∼10^2^ kPa, which is lower than that of PDMS (∼10^3^ kPa). Thus, the stretchable and compliant ionogel negligibly constrains the actuation and the area strain derived from soft PDMS. At 1 s, a voltage of 20 kV is applied and subsequently maintained at a constant level (as shown in the inset of Figure 5a). The actuator exhibits a rapid response, with a delay time of less than 1 s. Following this initial response, the area strain stabilizes and remains constant (Figure 5a). This response time is at the millisecond level, which is comparable to other reported actuators [45,46,47]. When the voltage was ramped up, the area strains were recorded until the dielectric elastomer failed due to electrical breakdown. A largest area strain of 36% was achieved when a 20 kV voltage was applied to the actuator (Figure 5a,b). This performance surpasses that of the hydrogel-based actuator, which exhibits an area strain of about 27% under a significantly lower driving voltage of 4 kV [48]. And the area strain–voltage curve of the ionogel-based actuator exhibits a high degree of similarity to that of hydrogel-based actuators. The good actuation performance can be attributed to the excellent mechanical properties and superior chemical stability of PAA ionogels. The electromechanical response of the actuator shows frequency dependence. When subjected to cyclic voltage, the actuator initially oscillates with a drifting actuation magnitude due to the viscoelastic nature of PDMS [49]. After several cycles, the actuator reaches a steady oscillation state. We plotted the steady-state area strain as a function of frequency (Figure 5d). The area strain decreases rapidly with increasing excitation frequency. The reason for this rapid reduction is unclear [50]. More systematic experiments and calculations are needed.

It is worth noting that the ionogels maintained their chemical stability throughout the actuation process. At the interface between the electronic conductor (aluminum) and the ionic conductor (ionogel), charges are separated over nanometer-scale distances. In contrast, charges on the two faces of the dielectric elastomer are separated by its thickness. As a result, the EDL has an enormous capacitance compared with the dielectric elastomer. This leads to a small voltage drop across the EDL, which prevents electrochemical reactions, while a larger voltage drop across the dielectric elastomer enables electromechanical transduction. Specifically, in our experiments, the EDL and the dielectric elastomer are in series. When voltage *U* is applied, both the EDL capacitor and the dielectric elastomer capacitor receive the same amount of charge, *Q*. To estimate the behavior of the circuit, we assume that both capacitors are linear: *Q* = *C*_EDL_ *U*_EDL_ and *Q* = *C*_DE_ *U*_DE_, where *C*_EDL_ is the capacitance of the EDL, and *C*_DE_ is the capacitance of the dielectric elastomer. The capacitance of the EDL is given by *C*_EDL_ = *c*_EDL_ *A*_EDL_, where *c*_EDL_ is the capacitance per unit area of the EDL, and *A*_EDL_ is the area of the EDL. The capacitance of the dielectric elastomer *C*_DE_ = *ε A*_DE_/*H*_DE_, where *ε* is the permittivity, *A*_DE_ is the area of the dielectric elastomer, and *H*_DE_ is the thickness of the dielectric elastomer. Thus, the voltage distribution between the EDL and the dielectric elastomer can be expressed as

$$\frac{U_{EDL}}{U_{DE}}=\frac{A_{DE}}{A_{EDL}}\times \frac{\varepsilon }{H_{ED}}\times \frac{1}{c_{EDL}}.$$

For representative values *A*_EDL_/*A*_DE_ ≈ 10^−2^, *ε* ≈ 10^−11^ F m^−1^, *c*_EDL_ ≈ 10^−1^ F m^−2^ [51], and *H*_DE_ ≈ 10^−4^ m, we find that *U*_EDL_/*U*_DE_ ≈ 1 × 10^−4^. Consequently, when the total applied circuit voltage reaches 20 kV (the pre-failure actuation threshold), the series-connected actuator circuit induces a voltage division effect. This results in a voltage drop across the dielectric elastomer that is below 20 kV, while the voltage drop across the EDL remains below 2 V, well under the 4.25 V electrochemical stability limit of the ionic liquid [52]. Such a voltage distribution effectively inhibits any electrochemical reactions. Consistent with this analysis, no evidence of electrochemical reactions was observed during the entire experimental process, in which the actuator operated until failure. Moreover, the observed cause of actuator failure was the breakdown of the PDMS dielectric elastomer material, rather than damage to the PAA ionogels. Therefore, the device is limited by the electrical breakdown of the dielectric elastomer rather than by electrochemical reactions at the EDL.

In the cycle stability test, a sinusoidal voltage with an amplitude of 15 kV and a frequency of 16 Hz was applied to the dielectric elastomer actuator. The area strain was measured and recorded every 10⁵ cycles, and the experiment was concluded after 10⁶ cycles, as depicted in Figure 5d. This cycle number is substantially higher than those reported in previous studies [53,54]. The actuator demonstrated remarkable durability and a long lifetime, maintaining the area strain for at least a million cycles without degradation. This robust performance is partly attributed to the excellent adhesion of the PAA ionogel. The bond between the PAA ionogel and PDMS is sufficiently strong to withstand the actuation forces, and no delamination was observed during operation. However, if the actuator operates over an extremely long period, the adhesion between layers may gradually decrease due to the influence of environmental humidity and temperature. This reduction in adhesion can lead to delamination of the actuator material, ultimately causing device failure. In comparison, fatigue damage resulting from long-term operation has a relatively minor impact. This is because the maximum area strain experienced by the actuator is significantly smaller than its high stretchability, and it can thus be considered as elastic deformation.

The high-performance actuator holds significant potential for diverse applications, including soft robotics, human–machine interaction, flexible displays, and energy harvesting. In soft robotics, it exhibits biomimetic actuation characteristics comparable to biological muscles, enabling applications in crawling, aerial, underwater, and jumping or rolling robotic systems [54]. For human–machine interaction, its integration into wearable textiles provides real-time responsiveness to both human motion and environmental stimuli, facilitating innovative applications such as adaptive massage systems and posture correction devices. Within flexible displays, the actuator can dynamically modulate screen shape or transparency via electric fields, facilitating advanced interactive visualization. Additionally, the actuator shows promise for emerging applications including vibration energy harvesting, adaptive window systems, and deformable aerodynamic surfaces.

## 3. Conclusions

In summary, we have successfully developed a transparent, stretchable, and adherable ionogel with mechanical properties closely resembling those of human skin through a simple one-step polymerization reaction. The PAA ionogels demonstrate remarkable properties, including high transparency (>98%), stretchability (with a largest fracture strain of ∼19), low hysteresis (<5.83%), strong adhesion, and a wide operating temperature range. By tuning the content of monomers and crosslinkers, the ionogels achieve a Young’s modulus that closely matches that of human skin, ranging from 1 to 103 kPa. Furthermore, the PAA ionogel was utilized as an ionic conductor to assemble a dielectric elastomer actuator, which demonstrated excellent actuation response and electrochemical stability. The voltage and frequency of the applied excitation are two key parameters influencing the area strain of the ionogel-based actuator. The actuator exhibits a fast response time (less than 1 s) and a large area strain of 36%, along with outstanding durability and reliability over millions of cycles. The study will prompt the use of ionogels as ionic conductive materials in stretchable devices and will further accelerate the development of soft electronics.

It should be noted that the ionic liquids incorporated in PAA-based ionogels may exhibit potential dermal irritation or toxicity upon skin contact. To address this safety concern, two practical mitigation strategies are proposed: (i) integrating ionogels into textile-based garments, or (ii) encapsulating them within biocompatible, flexible protective layers. These approaches would effectively prevent direct skin exposure while maintaining functional performance. Furthermore, our future research will focus on developing ionogel formulations using less toxic ionic liquid alternatives to minimize potential safety risks. These developments are expected to significantly enhance the practical applicability of ionogel-based electronic devices.

## 4. Materials and Methods

### 4.1. Materials

The monomer of acrylic acid (AA), the initiator of α-ketoglutaric acid, and the ionic liquid of 1-ethyl-3-methylimidazolium ethylsulfate ([C_2_mim][EtSO_4_]) were purchased from Shanghai Aladdin Biochemical Technology Co., Ltd. (Shanghai, China). The crosslinker N,N′-methylenebisacrylamide (MBAA) was purchased from Sigma-Aldrich LLC (St. Louis, MO, USA). The poly(dimethylsiloxane) (PDMS) precursor and curing agent (Sylgard 184) were purchased from Dow Corning (Midland, MI, USA). All reagents were used as received without further purification.

### 4.2. Preparation of PAA Ionogels

The ionogel was synthesized through a simple radical polymerization process. First, specific quantities of monomer AA, crosslinker MBAA, and initiator α-ketoglutaric acid were dissolved in the ionic liquid [C_2_mim][EtSO_4_]. This mixture was stirred for 30 min to form a homogeneous precursor solution. To precisely control the properties of the resulting ionogel, the concentrations of AA and MBAA were carefully adjusted. The concentration of AA ranged from 10% to 50% by weight relative to the ionic liquid, while the concentration of MBAA ranged from 0.2% to 3.0% by weight relative to AA. The initiator concentration was set at 0.5% by weight relative to AA. Once the precursor solution was prepared, it was carefully transferred and either poured into a glass cell (100 mm × 100 mm) separated by a silicone spacer (0.2 or 2 mm thick) or into a cylindrical mold. The solution was then exposed to UV irradiation at a wavelength of 365 nm and a power of 40 W for 4 h at room temperature, facilitating polymerization and yielding PAA ionogels. Finally, the PAA ionogels were accurately cut into desired shapes to enable precise evaluation of their performance and characteristics in various tests.

### 4.3. Mechanical Testing

Mechanical testing was performed using a mechanical testing machine (CMT6503, MTS, Minneapolis, MN, USA) equipped with a 100-N load cell. The samples were shaped into dumbbell forms using a standardized cutter, with specific dimensions: a total length of 35 mm, a width of 2 mm, and a gauge length of 12 mm. The dumbbell-shaped specimens were then firmly clamped at both ends in the testing machine and subjected to stretching at a consistent rate of 100 mm min^−1^. During this process, the stress–strain curves were recorded. For cyclic loading–unloading measurements, a more complex procedure was employed. The dumbbell-shaped samples were initially loaded up to a predetermined strain level, then unloaded back to zero strain. This loading–unloading cycle was repeated multiple times, all while maintaining the constant stretching rate of 100 mm min^−1^.

### 4.4. Transmittance Testing

The optical transparency of the ionogels was evaluated by measuring their transmittance using a high-precision spectrophotometer (UV-2250, Shimadzu Corporation, Kyoto, Japan). The measurements were conducted across the entire visible spectrum, ranging from 400 nm to 780 nm, which encompasses the wavelengths that are typically visible to the human eye.

### 4.5. TGA Testing

TGA was conducted to assess the thermal stability of the PAA ionogels. The tests were carried out using a Netzsch TG 209 instrument (Selb, Germany) under a nitrogen atmosphere. The temperature was ramped from 20 °C to 700 °C at a heating rate of 10 °C min^−1^. For each run, about 8 mg of ionogel sample was placed in an alumina crucible. Additionally, the ionic liquid was analyzed as a control.

### 4.6. Strain-Dependent Resistance Testing

The electrical resistance of the ionogels during stretching was evaluated using the four-point probe technique. In this setup, two outer probes were attached to the ends of a dumbbell-shaped ionogel specimen to complete an electrical circuit, with the current measured using a galvanometer (Agilent 34461A, Keysight Technologies, Santa Clara, CA, USA). Simultaneously, two inner probes were connected to the ends of the central gauge section of the dumbbell-shaped ionogel to measure the voltage with a voltmeter (VC8901A, Shenzhen Yisheng Victory Technology Co., Ltd., Shenzhen, China). The ionogel’s resistance was calculated by dividing the measured voltage by the current, a value that changes dynamically with the applied tensile strain.

### 4.7. Fabrication of Dielectric Elastomer Actuator

The PDMS film was pre-stretched to 1.5 times its original radius, resulting in a thickness of 0.1 mm, and then mounted onto a rigid acrylic ring with an inner diameter of 66.5 mm. Ionogel sheets with a thickness of 0.2 mm were cut into circular shapes with a diameter of 15.0 mm and equipped with two symmetrical strips (each 2 mm in width) using a laser cutting system (Versa Laser VLS2.30, Universal Laser Systems, Scottsdale, AZ, USA). Two circular ionogel membranes were carefully placed on either side of the pre-stretched PDMS sheet to form a sandwich structure. Each ionogel membrane was connected, via the symmetrical strips, to aluminum tape that was affixed to a rigid frame linked to a power source (Model 30/20, TREK, Waterloo, IA, USA).

### 4.8. Area Strain Testing of Dielectric Elastomer Actuator

A video camera (ILCE-7M4, Sony, Kyoto, Japan) was employed to record the lateral expansion of the actuators as the voltage was incrementally applied in 1 kV steps. At each voltage level, the voltage was maintained for 10 s to ensure stable area strains were achieved. The captured video images were analyzed frame by frame using image analysis software (ImageJ 1.x) to determine the change in the overlapping area between the top and bottom electrodes. The area strain values (%) were calculated by

$$\mathrm{area}\;\mathrm{strain}\;(\%)=\frac{A_{t}-A_{0}}{A_{0}}\times \;100\%,$$

where *A*_t_ represents the area in the actuated state, and *A*_0_ is the initial area. Area strain–voltage curves were plotted as the voltage increased. The frequency dependence of area strain was measured for the actuator at a voltage of 15 kV. When the voltage was cycled at a specific frequency, the actuator oscillated. The area strain of the actuator was recorded over a frequency range of 0.06 Hz to 128 Hz after the actuator reached a steady oscillation. For cycle stability measurements, the actuators were subjected to a voltage of 15 kV at a frequency of 16 Hz. The area strain was recorded after every 10^5^ cycles, and the experiment was terminated after 10^6^ cycles.

## Data Availability

The original contributions presented in the study are included in the article; further inquiries can be directed to the corresponding author.
